# A new species and additional records of *Lobrathium* Mulsant & Rey (Coleoptera, Staphylinidae, Paederinae) from South China

**DOI:** 10.3897/zookeys.568.7622

**Published:** 2016-02-23

**Authors:** Zhong Peng, Li-Zhen Li, Mei-Jun Zhao

**Affiliations:** 1 Department of Biology, College of Life and Environmental Sciences, Shanghai Normal University, Shanghai, 200234, P. R. China

**Keywords:** Coleoptera, Staphylinidae, *Lobrathium*, new species, new records, China

## Abstract

Material of the genus *Lobrathium* Mulsant & Rey, 1878 from the Chinese provinces Fujian, Hunan, Sichuan, Guangdong and Guangxi is examined. Six species are identified, four of them described previously and two undescribed. *Lobrathium kedian* Peng & Li, **sp. n.** (Guangxi: Shiwangda Shan) is described and illustrated. One probably undescribed species remains unnamed. The female sexual characters of *Lobrathium flexum* Assing, 2014 are described and illustrated for the first time. The genus is now represented in mainland China by 43 species.

## Introduction

Until today, 42 species of the genus *Lobrathium* Mulsant & Rey have been reported from mainland China and 20 species from Taiwan (Assing 2010, 2012, 2013, 2014; Li et al. 2013; Li et al. 2013a, b, c; Lü and Li 2014). With a total of 14 described species, the *Lobrathium* fauna of Sichuan is currently more diverse than that of any of the other Chinese provinces, followed by Yunnan (8 species), Shaanxi (7 species), Guizhou (6 species) and Zhejiang (6 species) (Assing 2012, 2013, 2014; Li et al. 2013; Li et al. 2013a, b, c; Lü and Li 2014).

A study of *Lobrathium* material from southern China yielded a species new to science and additional records of *Lobrathium configens* Assing, 2012, *Lobrathium flexum* Assing, 2014, *Lobrathium hebeatum* Zheng, 1988 and *Lobrathium hongkongense* Bernhauer, 1931.

## Material and methods

The following abbreviations are used in the text, with all measurements in millimeters:

Body length (BL) from the anterior margin of the labrum to the abdominal apex; forebody length (FL) from the anterior margin of the labrum to the posterior margin of the elytra; head length (HL) from the anterior clypeal margin to the occipital constriction; head width (HW): maximum width of head; length of antenna (AnL); length of pronotum (PL) along midline; maximum width of pronotum (PW); elytral length (EL) at the suture from the apex of the scutellum to the posterior margin of the elytra (at the sutural angles); maximum width of the elytra (EW); length of aedeagus (AL) from the apex of the dorsal plate to the base of the aedeagal capsule.

The type material is deposited in the Insect Collection of Shanghai Normal University, Shanghai, China (SNUC).

## Results

### Lobrathium configens

Taxon classificationAnimaliaColeopteraStaphylinidae

Assing, 2012

Fig. 4

#### Material studied.

China: Sichuan: 8 ♂♂, 2 ♀♀, Xiaojin County, Jiajin Shan, 30°48'49"N, 102°42'55"E, 2500 m, 20.VII.2015, Jiang, Peng, Tu & Zhou leg. (SNUC).

#### Comment.

*Lobrathium configens* was previously known from the Chinese provinces Shaanxi, Sichuan, Qinghai, Hubei, Yunnan and Zhejiang (Assing 2012, 2013, 2014; Li et al. 2013a, b). For illustrations of *Lobrathium configens* see Assing (2012: figures 153–165) and Li et al. (2013a: figure 4).

### Lobrathium flexum

Taxon classificationAnimaliaColeopteraStaphylinidae

Assing, 2014

Figs 1A , 2A–C , 5

#### Material studied.

China: Hunan: 1 ♂, 1 ♀, Yanling County, Nanfengmian, 26°18'N 114°00'E, 1600 m, 06.VI.2015, Peng, Shen, Tu & Zhou leg. (SNUC).

#### Comment.

The original description is based on a male from Jiangxi. The previously unknown female sexual characters are as follows: posterior margin of tergite VIII convex (Fig. 2A); sternite VIII (Fig. 2B) weakly transverse, posteriorly broadly convex; tergite IX (Fig. 2C) undivided anteriorly. The above record from Hunan represents a new province record. For illustrations of the habitus and the male sexual characters see Assing (2014: figures 12–16).

**Figure 1.**
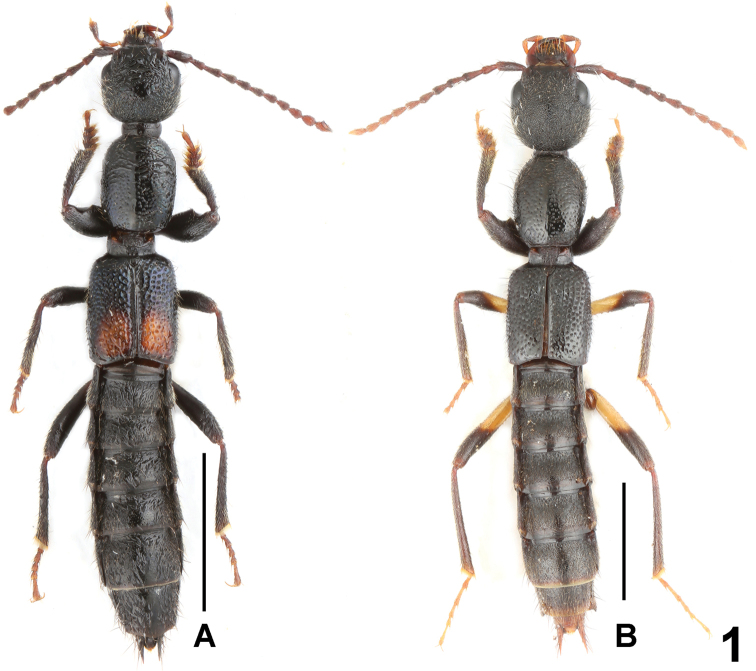
Habitus: **A** *Lobrathium flexum* **B** *Lobrathium kedian*. Scale bars: 2.0 mm.

**Figure 2.**
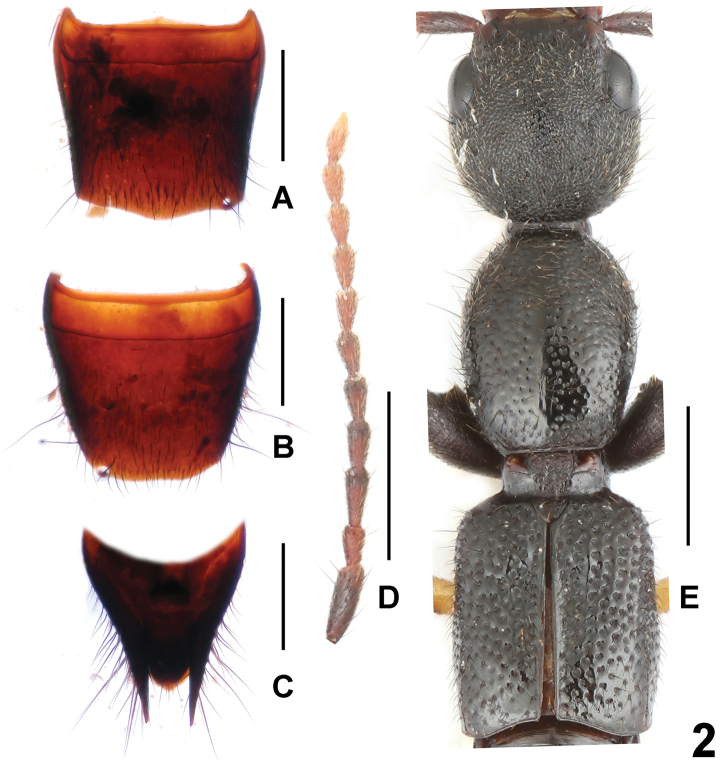
*Lobrathium flexum* (**A–C**) and *Lobrathium kedian* (**D–E**). **A** female tergite VIII **B** female sternite VIII **C** female tergites IX–X **D** antenna **E** forebody. Scale bars: 0.5 mm (**A–C**), 1.0 mm (**D–E**).

### Lobrathium hebeatum

Taxon classificationAnimaliaColeopteraStaphylinidae

Zheng, 1988

Fig. 6

#### Material studied.

China: Sichuan: 1 ♂, 3 ♀♀, Dayi County, Xiling Xueshan, 30°41'59"N, 103°12'10"E, 2150 m, 29.VII.2015, Jiang, Peng, Tu & Zhou leg. (SNUC).

#### Comment.

The previously known distribution of *Lobrathium hebeatum* included the Chinese provinces Shaanxi, Sichuan, Yunnan, Henan and Ningxia (Assing 2012, 2013, 2014; Li et al. 2013a, b; Zheng 1988). For illustrations of *Lobrathium hebeatum* see Assing (2012: figures 142–147) and Li et al. (2013a: figure 9).

### Lobrathium hongkongense

Taxon classificationAnimaliaColeopteraStaphylinidae

Bernhauer, 1931

Figs 5 , 8

#### Material studied.

China: Fujian: 3 ♂♂, Nanping, Mangdang Shan, 26°41'51"N, 118°07'00"E, 400 m, 10.IX.2015, Yan & Tang leg. (SNUC). Hunan: 2 ♂♂, 1 ♀, Yanling County, Nanfengmian, 26°18'N 114°00'E, 1600 m, 06.VI.2015, Peng, Shen, Tu & Zhou leg. (SNUC). Guangdong: 1 ♂, 1 ♀, Ruyuan County, Nanling Nature Reserve, Qingshuigu, 24°54'57"N, 113°01'55"E, 900 m, 04.V.2015, Peng, Tu & Zhou leg. (SNUC); 1 ♀, Jieyang, Puning, Wufeng Shan, 500 m, 08.VI.2015, Aranyu leg. (SNUC).

#### Comment.

*Lobrathium hongkongense* was previously known from Japan, Hong Kong, Taiwan and the Chinese provinces Fujian, Guizhou, Zhejiang, Jiangsu, Sichuan, Yunnan, Guangxi, Hubei and Shaanxi (Assing 2012, 2013; Li et al. 2013a, b). The specimens represent the first record from Hunan and Guangdong. For illustrations of *Lobrathium hongkongense* see Assing (2012: figures 125–132) and Li et al. (2013a: figure 10).

### Lobrathium kedian

Taxon classificationAnimaliaColeopteraStaphylinidae

Z. Peng & L.-Z. Li sp. n.

http://zoobank.org/546F9FE5-C3BA-469F-A96A-8FC2117C7634

Figs 1B , 3 , 7

#### Type material.

Holotype: ♂, labelled ‘China: Guangxi Prov., Shangsi County, Shiwanda Shan, 300–500 m, 21°54'N, 107°54'E, 25–IV–2011, Peng & Zhu leg.’ (SNUC). Paratypes: 8 ♂♂, 8 ♀♀, same label data as holotype (SNUC).

#### Description.

Measurements (in mm) and ratios: BL 9.88–10.20, FL 5.49–5.62, HL 1.37–1.42, HW 1.36–1.44, AnL 3.13–3.20, PL 1.57–1.63, PW 1.24–1.30, EL 1.35–1.39, EW 1.39–1.46, AL 1.13–1.20, HL/HW 0.97–1.00, HW/PW 1.06–1.10, HL/PL 0.85–0.88, PL/PW 1.25–1.27, EL/PL 0.84–0.87.

Habitus as in Fig. 1B. Coloration: body black, mandibles dark brown, labial palpi light brown; antennae dark brown to light brown; legs with blackish brown profemora and protibiae, basal halves of meso- and metafemora yellowish brown, distal halves gradually infuscate.

Head as wide as long, widest behind eyes; punctation coarse and very dense; interstices without microsculpture. Antenna as in Fig. 2D.

Pronotum distinctly longer than wide, with impunctate midline; punctation coarse and dense, but distinctly sparser than that of head; interstices glossy.

Elytra distinctly broader than pronotum; punctation coarse, arranged in irregular series only laterally. Hind wings approximately 1.85–2.02 times as long as elytra.

Abdomen somewhat narrower than elytra; punctation fine and dense; posterior margin of tergite VII with palisade fringe.

**Figure 3.**
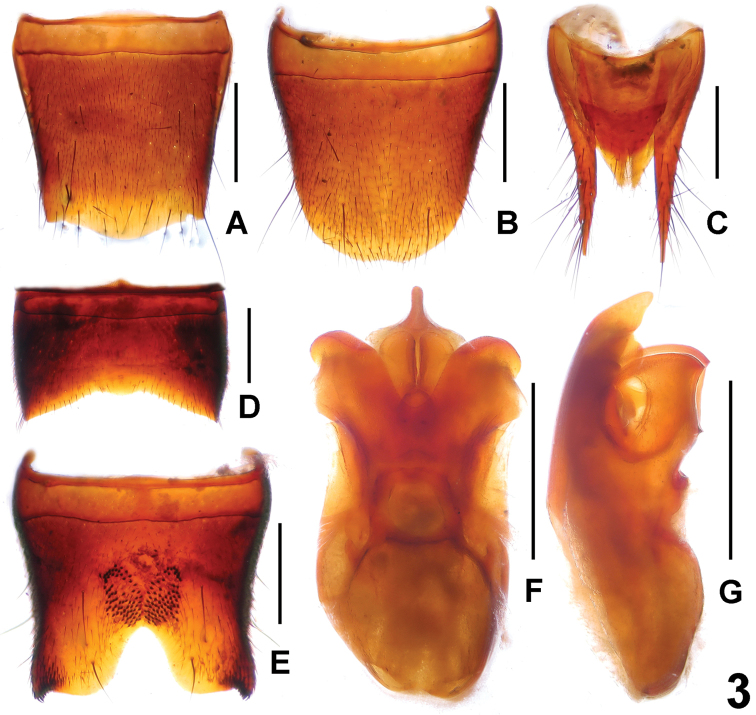
*Lobrathium kedian*. **A** female tergite VIII **B** female sternite VIII **C** female tergites IX–X **D** male tergite VIII **E** male sternite VIII **F** aedeagus in ventral view **G** aedeagus in lateral view. Scale bars: 0.5 mm.

Male. Sternite VII (Fig. 3D) strongly transverse and with shallow median impression posteriorly, without modified setae, posterior margin broadly concave; sternite VIII (Fig. 3E) posteriorly with deep impression, this impression with a cluster of numerous short peg-setae, postero-laterally with a cluster of short black setae; posterior excision large, deep and U-shaped; aedeagus (Figs 3F, G) with apically bifid ventral process in ventral view and broad dorsal plate.

Female. Posterior margin of tergite VIII (Fig. 3A) convex; sternite VIII (Fig. 3B) weakly transverse, posterior margin broadly convex; tergite IX (Fig. 3C) slender and undivided anteriorly.

#### Distribution and natural history.

The type locality is situated in Shiwangda Shan to the south of Shangsi, southern Guangxi. The specimens were sifted from leaf litter in broad-leaved forests at altitudes of 300–500 m (Fig. 7).

**Figures 4–8.**
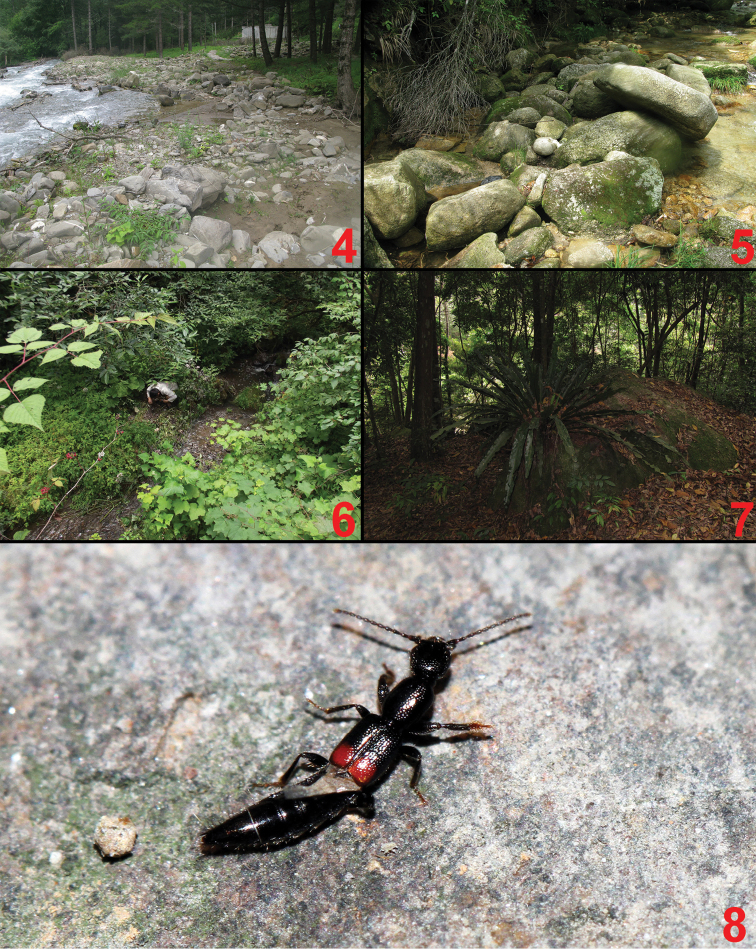
Habitats of *Lobrathium*. **4** Jiajin Shan, alt. 2500 m (*Lobrathium configens*) **5** Nanfengmian, alt. 1600 m (*Lobrathium flexum* and *Lobrathium hongkongense*) **6** Xiling Xueshan, alt. 2150 m (*Lobrathium hebeatum* and *Lobrathium* sp.) **7** Shiwangda Shan, alt. 300–500 m (*Lobrathium kedian* sp. nov.) **8** *Lobrathium hongkongense* walking on the stone.

#### Etymology.

The specific name is the Chinese noun “kedian” (punctation) in apposition. It refers to the punctation of the head of *Lobrathium kedian*, which is denser than that of other species known from Guangxi.

#### Comparative notes.

*Lobrathium kedian* shares a bifid ventral process with *Lobrathium digitatum* Assing, 2010 from Taiwan, but differs from it in many respects, particularly by larger body size, the shape and chaetotaxy of the male sternite VIII and by the shape of the aedeagus. For illustrations of *Lobrathium digitatum* see Assing (2010: figures 203–210).

### Lobrathium

Taxon classificationAnimaliaColeopteraStaphylinidae

sp.

Fig. 6

#### Material studied.

China: Sichuan: 1 ♀, Dayi County, Xiling Xueshan, 30°41'59"N, 103°12'10"E, 2150 m, 29.VII.2015, Jiang, Peng, Tu & Zhou leg. (SNUC).

#### Comment.

This species is similar and probably closely related to *Lobrathium daxuense* Assing, 2012. The female represents an undescribed species distinguished from its congeners particularly by the light brown coloration, large body size (8.34 mm), much denser punctation of the head, a slender pronotum, and the female secondary sexual characters.

## Supplementary Material

XML Treatment for Lobrathium configens

XML Treatment for Lobrathium flexum

XML Treatment for Lobrathium hebeatum

XML Treatment for Lobrathium hongkongense

XML Treatment for Lobrathium kedian

XML Treatment for Lobrathium
